# The importance of calcium in improving resistance of *Daphnia* to *Microcystis*

**DOI:** 10.1371/journal.pone.0175881

**Published:** 2017-04-17

**Authors:** Siddiq Akbar, Jingjing Du, Yong Jia, Xingjun Tian

**Affiliations:** 1 School of Life Science, Nanjing University, Nanjing, China; 2 Henan Collaborative Innovation Center of Environmental Pollution Control and Ecological Restoration, School of Material and Chemical Engineering, Zhengzhou University of Light Industry, Zhengzhou, China; 3 Jiangsu Key Laboratory for Microbes and Functional Genomics, Jiangsu Engineering and Technology Research Center for Industrialization of Microbial Resources, College of Life Sciences, Nanjing Normal University, Nanjing, China; VIT University, INDIA

## Abstract

Changing environmental calcium (Ca) and rising cyanobacterial blooms in lake habitats could strongly reduce *Daphnia* growth and survival. Here, we assessed the effects of maternal Ca in *Daphnia* on transfer of resistance to their offspring against *Microcystis aeruginosa* PCC7806 (*M*. *aeruginosa*). Laboratory microcosm experiments were performed to examine effects in *Daphnia carinata* (*D*. *carinata*) and *Daphnia pulex* (*D*. *pulex*), and that how Ca induce responses in their offspring. The results showed that growth and survival were increased in offspring from exposed *Daphnia* as compared to unexposed, when raised in high Ca and increasing *M*. *aeruginosa* concentration. Among exposed *Daphnia*, offspring from high Ca mothers, produced more neonates with large size and higher survival as compared to offspring from low maternal Ca. Exposed *D*. *carinata* and *D*. *pulex* offspring, when reared in Ca deficient medium and increasing *M*. *aeruginosa* concentration, time to first brood increased, size become large and total offspring decreased subsequently in three alternative broods in offspring from low maternal Ca. In contrast, growth and reproduction in offspring from high Ca exposed mothers were consistent in three alternative broods. Despite species specific responses in growth, survival and variant life history traits in two *Daphnia* species, our results not only show maternal induction in *Daphnia* but also highlight that offspring response to *M*. *aeruginosa* varies with maternal Ca. This study demonstrates that Ca have role in *Daphnia* maternal induction against *Microcystis*, and recent Ca decline and increasing *Microcystis* concentration in lakes may decrease *Daphnia* growth and survival. Our data provide insights into the interactive effect of maternal Ca and *Microcystis* exposure on *Daphnia* and their outcome on offspring life history traits and survival.

## Introduction

Zooplankton plays an essential role in transfer of energy to higher trophic levels [1]. Phytoplankton abundance in aquatic environment is restrained by zooplankton grazing, which is necessary for balanced ecosystem [2, 3]. However, recently aquatic pelagic ecosystem is dominated by cyanobacteria [4]. Cyanobacteria (e.g. *Microcystis*) display high resistance to grazing [5], which often leads to reduced growth and reproduction of predators [6]. The inability of zooplankton to graze on cyanobacteria is traditionally linked to inadequate food source due to their toxicity, low nutritional value and production of large colonies [7–9]. In order to cope with cyanobacterial toxicity, zooplankton develop several counter defences to enhance their success during cyanobacterial blooms [10, 11]. *Daphnia* due to its large size is considered an important zooplankton with the highest potential to control blooms [11]. Recently maternal induction has been investigated in several *Daphnia* species and clones [12–14]. Maternal effects are expressions of offspring phenotype that arise through an interaction between the maternal genotype and the environment [6]. Stimuli experienced by parents can be transmitted into offspring to make them more resistant under adverse conditions. Prior work has shown that exposure of *Daphnia* to toxic strain can enhance fitness of their offspring [10]. Similarly, Jiang et al [13] observed that exposed *Bosmina longirostris* clones enhance resistant against toxic *M*. *aeruginosa* as compared to unexposed.

One aspect of maternal environment that could affect an offspring phenotype is food. In *Daphnia*, maternal food concentration has been found to affect juvenile survival, growth, reproduction and longevity [15]. Maternal exposure to predator kairomones enhanced offspring defenses [16], and an interaction between maternal food quantity and photoperiod impact offspring resting-egg production [17].

Food enriched with certain nutrients has the potential to alter competitive interactions among consumers, and has been shown to have profound effects on the diversity and the composition of zooplankton communities [18]. The lipid content and fatty acid composition of the maternal diet has been shown to affect reproductive strategies and the biochemical composition of the eggs [19], and the performance of offspring in *Daphnia* [15]. Subsequent research indicated that *D*. *pulicaria* isolated from high-nutrient lakes were less inhibited by toxic cyanobacteria than clones isolated from low-nutrient lakes [11]. Jiang, Li [13] observed that maternal effects of inducible defenses against cyanobacteria differ among clones in one species of daphnids and activation of maternal effects require additional resources. Similarly, Mckee and Ebert [20] showed that maternal food quantity changed the effect of maternal temperature on offspring size in *D*. *magna*.

Inadequate intake of certain elements, such as N and P, strongly affects key physiological processes in many animals [21, 22]. Likewise, Ca is also vital for zooplankton and affect various physiological processes such as formation of structural components of carapaces and molting [23, 24]. The major route of Ca for Crustacean zooplankton is active transport from surrounding water [25]. *Daphnia* molt frequently and has no Ca storage potential [26]. The majority of their Ca must be taken from the external environment immediately after molting [27, 28]. Recently, Ca decline in soft-water lakes has negatively affected the keystone zooplankter *Daphnia* in Canadian Shield, North America and Scandinavian countries with critical implication for aquatic food web [29]. Ca could be a limiting factor for certain species in soft-water localities, especially for those species that have high Ca requirements for their carapaces. It has been investigated that low Ca conditions greatly increase the metabolic costs of individuals and natural food limitation increased Ca requirements in the field [30].

Ca decline in soft-water lakes commonly co-occur with other anthropogenic and natural stressors, which may cause unexpected and complex interactions [31]. Ca decline weaken the phosphate-binding potential of lake sediments, thereby increasing the release of internal phosphate and causing the dominance of cyanobacteria. In addition, cyanobacteria dominate some soft-water lakes due to acidic deposition and deforestation in watersheds [32]. Alternatively, Ca deficiency restricts the proliferation of Ca-rich *Daphnia*, which can exert strong grazing pressure on algae [33]. *D*. *magna and D*. *tenebrosa* were susceptible to UV radiation, and susceptibility for both species increased significantly with decreased Ca content [27]. Increasing cyanobacteria and Ca deficiency significantly decrease fitness in the large-bodied *D*. *pulex*, while warming did not [13]. Recently, Riessen, Linley [34] analyzed that low aqueous Ca (<1.5mg/L) in *D*. *pulex* block the kairomones-induced development of adaptive defense and increase vulnerability.

The role of *Daphnia* maternal induction in the adaptation of their offspring to changing environment is intriguing. Maternal induction has been studied in controlled conditions in several *Daphnia* species, and offspring of exposed mothers showed enhanced resistance to *Microcystis* [35, 36]. Frost, Ebert [37] investigated that *D*. *magna* transferred phosphorous stress to their offspring. Similarly, Sperfeld and Wacker [15] suggested that maternal diets should be deliberately varied in future studies to assess ecological relevant food quality effects on zooplankton. On the basis of growing evidence that *Daphnia* are negatively influenced by *Microcystis* blooms and Ca decline, the possibility that maternal Ca may alter offspring growth and survival in increasing amount of *Microcystis* has never been tested. Therefore, we test the hypothesis that (i) Ca may regulate maternal effects in *Daphnia* against *Microcystis* and high maternal Ca could increase growth and reproduction, (ii) mothers having high Ca concentration will efficiently allocate resources for growth, and inducible defenses may persist long in their offspring as compared to low Ca

## Materials and methods

### Cultures conditions

The microcystin-producing cyanobacterium *M*. *aeruginosa* (FACHB-915), and the green alga *Chlorella vulgaris* (*C*. *vulgaris*) (FACHB-1227) were provided by the Freshwater Algae Culture Collection of the Institute of Hydrobiology (FACHB), Chinese Academy of Sciences, Wuhan, China. They were mass cultured separately in BG-11 medium at 25°C under a light intensity of approximately 50 μmol m^-2^ s^-1^ with a 12: 12 h light: dark cycle [38]. The log phase algae were harvested by centrifugation for 10–12 min at 3300 rpm.

The cladoceran *D*. *carinata* was originally isolated from Huaihe River Basin (N32°250–33°290 and E113°190–115°330) and grown in our lab, while *D*. *pulex* was provided by School of Environment, Nanjing University, and originally isolated from Taihu Lake (119°52'32'' -20°36'10'' E). *D*. *carinata* and *D*. *pulex* are common species and have been investigated for inducible defenses in their offspring against cyanobacteria and predators [13, 34, 35]. The stock cultures of *D*. *carinata* and *D*. *pulex* were raised in COMBO medium [39], and fed with *C*. *vulgaris* (1.5x10^5^ cells mL^-1^) every other day. The cultures were kept in a constant temperature chamber at 22±1.2°C at 50 μmol m^-2^ s^-1^ with a 12: 12 h light: dark cycle. These animals were maintained under these conditions for at least one month and served as mothers for the animals used in the experiments. All neonates for a given experiment were taken from a single brood and within 12–15 h after birth.

### Experiment 1

The first experiment was designed to verify maternal effects of inducible tolerance in *Daphnia* to *M*. *aeruginosa*, and that how maternal Ca alters this inducible tolerance in their offspring. This was achieved by exposing mothers to different feeding regimes and raising their offspring in an optimal standard conditions.

### Experiment 2

In second experiment, offspring from exposed mothers were taken and grown in Ca deficient medium. The purpose was to evaluate the effects of maternal Ca on induced tolerance against *M*. *aeruginosa*, and their persistence in three alternative broods.

### Experimental procedure

**Fig 1:**
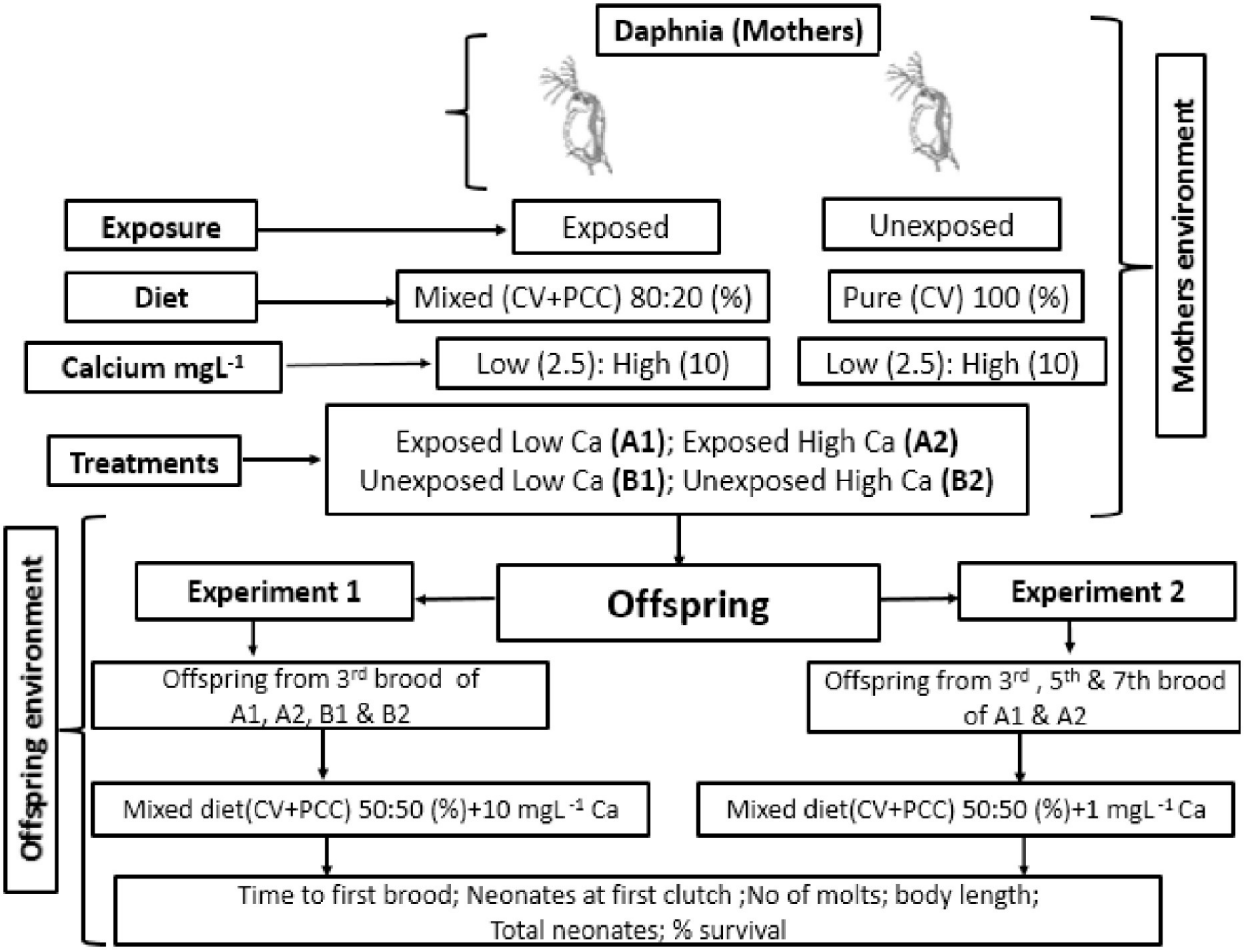
Scheme illustrating the experimental design of the current study.

*D*. *carinata* and *D*. *pulex* (F0) were individually exposed to mixed diet of *M*. *aeruginosa* (20%) and *C*. *vulgaris* (80%) (exposed) and or fed with pure *C*. *vulgaris* (unexposed) at low (2.5 mg L^-1^) and high (10 mg L^-1^) Ca as: (1) exposed *Daphnia* & low Ca: mixed diet with 2.5 mg L^-1^ Ca (A1); (2) exposed *Daphnia* & high Ca: mixed diet with 10 mg L^-1^ Ca (A2); (3) unexposed *Daphnia* & low Ca: pure diet with 2.5 mg L^-1^ Ca (B1); (4) unexposed *Daphnia* & high Ca: pure diet with 10 mg L^-1^ Ca (B2) as shown in Fig 1. Offspring (F1) of third brood were taken from four treatments within 12–15 h. They were raised on mixed diet of *M*. *aeruginosa* and *C*. *vulgaris* (50% each, 1 mg L^-1^ C) in full COMBO medium. Each treatment consist of ten replicates of *D*. *carinata* and *D*. *pulex* (F1). They were grown individually in 100 ml beaker having 60 ml COMBO medium. Different parameters such as, time to first brood, offspring produce at first clutch, body length, number of molts and total number of offspring were measured for three weeks. Neonates from four treatments were counted separately every day from first reproduction to day 21, and removed. The medium were replaced every other day. The body length of five *D*. *carinata* and *D*. *pulex* that were randomly taken from each treatment was measured. The body length was recorded as the distance from the top of the head to the base of the tail spine. These measurements were made using Gaosuo USB Digital Microscope 500X, China, calibrated with 0.1 mm gradations.

The second experiment was performed by taking offspring (F1) of 3rd brood (Fa) and two alternative broods, 5^th^ (Fb) and 7^th^ (Fc) (12–15 h old) from A1 and A2 treatments (Fig 1, Experiment 2) and grown in Ca deficient COMBO medium (1 mg L^-1^). All life history parameters and procedures were determined as mentioned in experiment 1.

Every 24 h during 3 weeks, survival of offspring (initial n = 10) were registered, and released juveniles were removed in each treatment.

### Statistical analysis

The phenotypic changes were analyzed as character state changes in the form of different parameters. Samples were taken and tested for differences in means of time to first brood, neonates produce at first clutch, molting, body length and total offspring produced among different treatments by two-way ANOVA and generalized linear model (GLM) for experiment 1 and experiment 2 respectively. A Levene’s test was conducted to determine normality. Further results were analyzed by multiple comparisons and Post Hoc Tukey’s tests. Differences in survival rates between treatments were tested with the Mantel Cox log-rank test. All statistical analysis were performed in SPSS package service version 16.

## Results

### Offspring growth and reproduction

**Fig 2:**
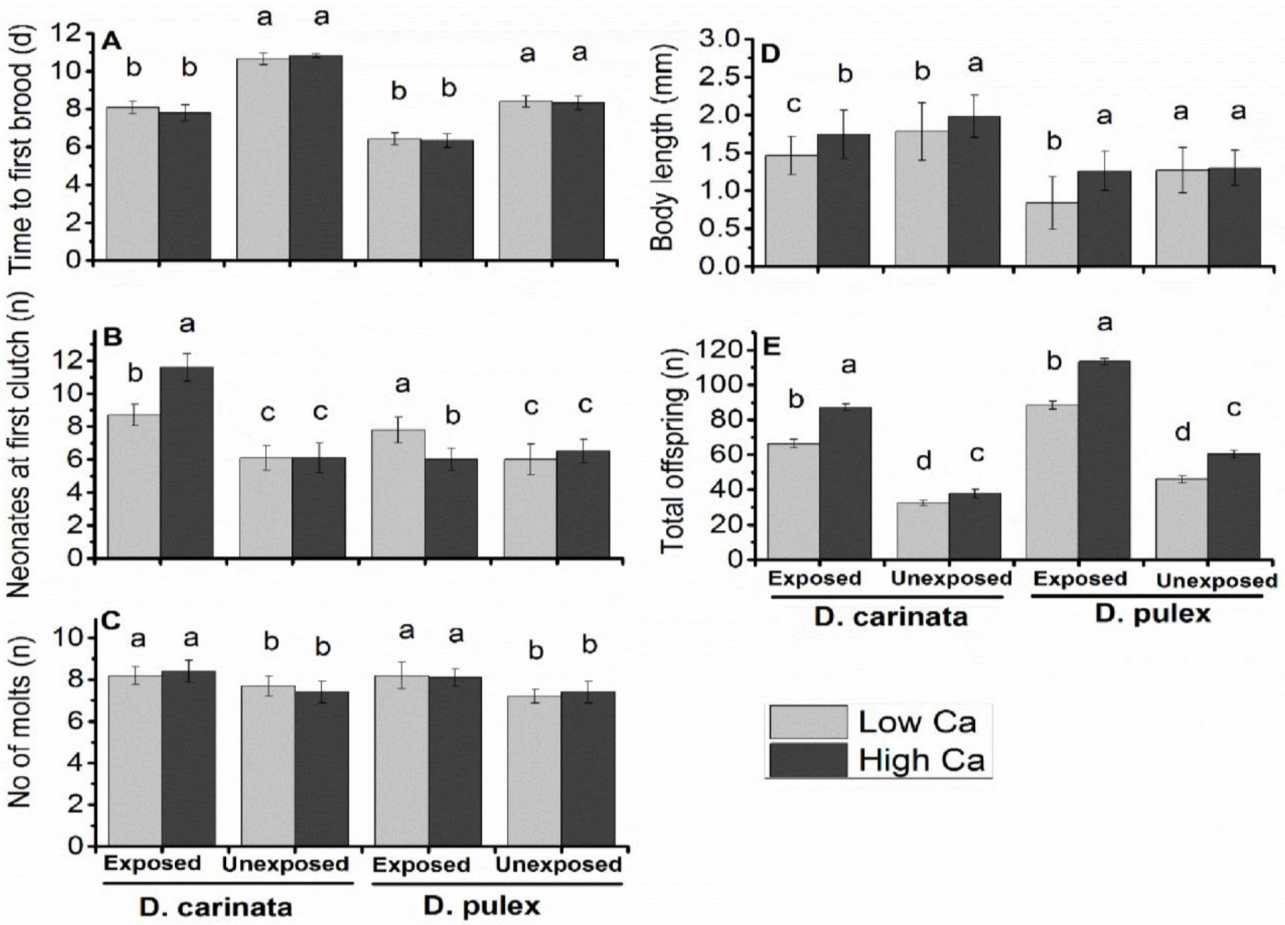
Life history parameters of offspring (F1) in Experiment 1. Note: (a), time to first brood; (b), neonates at first clutch; (c), number of molts (d), body length (e), total offspring. Different letters indicate significant difference (*p*<0.05) between neonates from exposed and unexposed treatments at low and high Ca. Error bars indicate ±SD, (n = 10).

We observed significant effect of maternal Ca and *M*. *aeruginosa* exposure on offspring growth and reproduction (Fig 2A–2E; Table 1). Offspring from exposed *D*. *carinata* and *D*. *pulex* produced neonates 2–3 days earlier, with large size and population increase by 20–25%, than their conspecifics with unexposed mothers (Fig 2A–2E). Among exposed mothers, offspring from high maternal Ca produced more neonates and large in size than those from low maternal Ca (Fig 2D and 2E; *p*<0.05). Neonates produced at first clutch from exposed *D*. *carinata* having high Ca was significantly more than that produced from offspring of low maternal Ca, while *in D*. *pulex* opposite trend was observed (Fig 2B; *p*<0.05). Neonates produced at first clutch was not significantly different between offspring from both low and high maternal Ca of unexposed *D*. *carinata* and *D*. *pulex* (Fig 2B). Number of molts produced in offspring of exposed *D*. *carinata* and *D*. *pulex* were significantly higher than unexposed (Fig 2C; *p*<0.05), while there was no significant difference in the number of molts between low and high maternal Ca in any of the studied cases (Fig 2C).

**Table 1:** Summary table of the two-way analyses of variance for Maternal exposure (Mat.exp), Maternal Calcium (Mat.Ca) and their interactive effects on *Daphnia* maternal induction against *Microcystis*.

Life history variables	Source of variation	*Daphnia* species	df	F	*p*
**Time to first brood (d)**	Mat.exp	DC	1	944.6	**0.001**
		DP	1	361.1	**0.001**
	Mat.Ca	DC	1	6.12	**0.018**
		DP	1	0.739	0.396
	Mat. exp x Mat. Ca	DC	3	0.366	0.549
		DP	3	0.009	0.924
**Neonates at first clutch (n)**	Mat.exp	DC	1	243	**0.001**
		DP	1	44.3	**0.001**
	Mat.Ca	DC	1	31.14	**0.001**
		DP	1	1.99	0.166
	Mat. exp x Mat. Ca	DC	3	31.14	**0.001**
		DP	3	0.009	0.924
**Number of molts (n)**	Mat.exp	DC	1	23.8	**0.001**
		DP	1	2.92	0.096
	Mat.Ca	DC	1	0.106	0.747
		DP	1	0.103	0.745
	Mat. exp x Mat. Ca	DC	3	2.64	0.112
		DP	3	19.75	0.335
**Body length (mm)**	Mat.exp	DC	1	156.8	**0.001**
		DP	1	8.4	**0.001**
	Mat.Ca	DC	1	115.2	**0.001**
		DP	1	9.3	**0.018**
	Mat. exp x Mat. Ca	DC	3	3.2	0.093
		DP	3	5.12	**0.04**
**Total offspring (n)**	Mat.exp	DC	1	4100	**0.001**
		DP	1	528	**0.001**
	Mat.Ca	DC	1	396	**0.001**
		DP	1	909.6	**0.001**
	Mat. exp x Mat. Ca	DC	3	142.1	**0.001**
		DP	3	65.8	**0.001**

df, degrees of freedom; F, Statistic for ANOVA test; *p*, significance of the ANOVA test.

**Fig 3:**
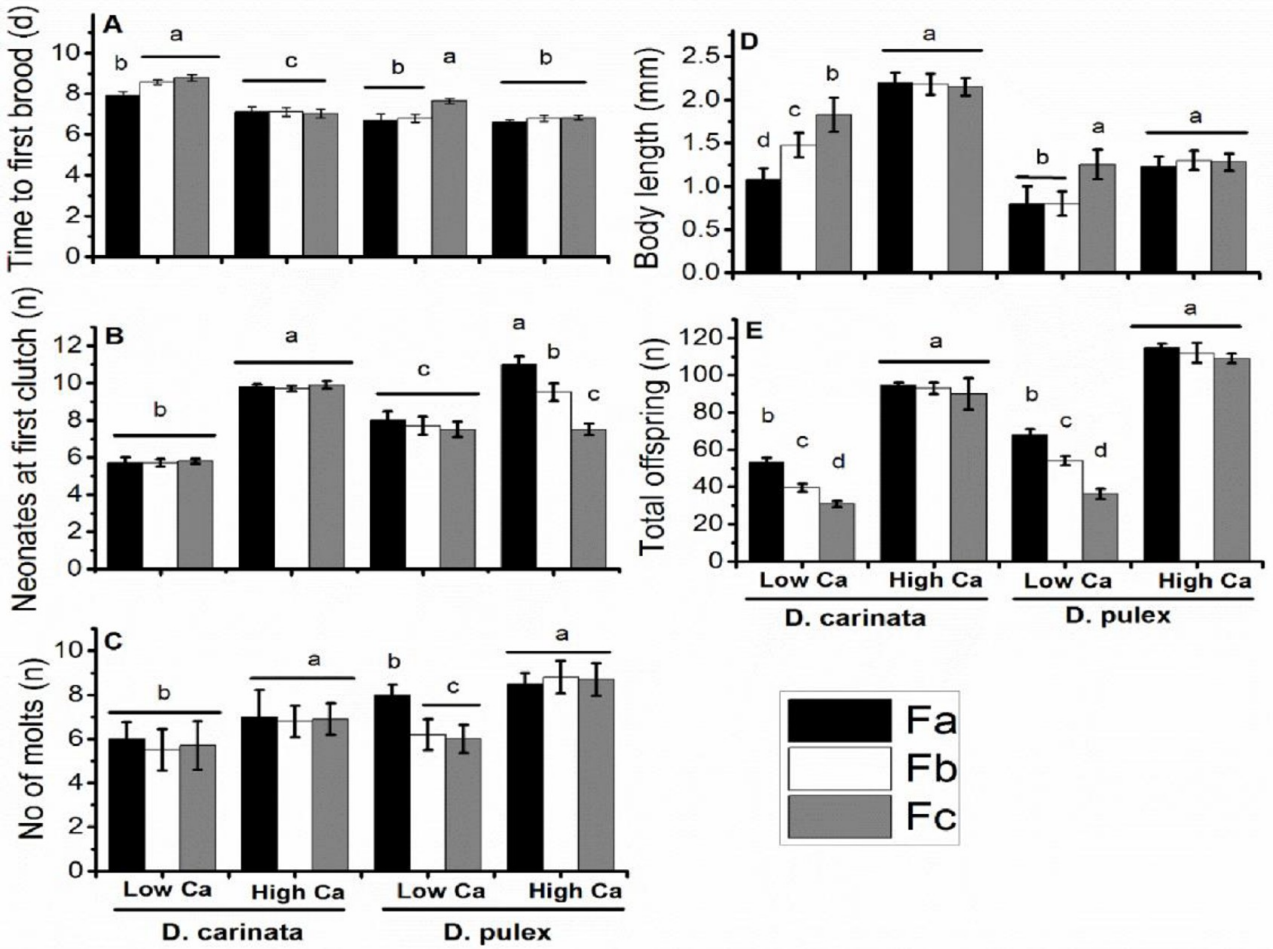
Life history parameters of three alternative broods of offspring (F1) in Experiment 2. Note: (a), time to first brood; (b), neonates at first clutch; (c), number of molts, (d) body length; (e), total offspring. Different letters indicate significant difference (*p*<0.05) between neonates from mothers exposed at low and high Ca. Error bars indicate ±SD, (n = 10). Fa, Fb and Fc represents third, fifth and seventh brood respectively.

Life history traits in offspring from *D*. *carinata* having high maternal Ca were not significantly different among three broods, except number of molts, but there were significant intraclutch variations in offspring from low maternal Ca (Fig 3A–3E). In case of offspring from *D*. *pulex* having high maternal Ca, neonates produced at first clutch and total offspring produced were significantly different among three broods (Fig 3B and 3E). When exposed in Ca deficient medium, time to first brood of offspring of *D*. *carinata* and *D*. *pulex* from low maternal Ca was prolonged to (0.7–0.9) day in Fb and Fc as compared to Fa brood, while it remain almost same in offspring of high maternal Ca (Fig 3A; *p*<0.05). Neonates produced at first clutch in *D*. *carinata* were significantly decreased in offspring from low maternal Ca (Fig 3B; *p*<0.05), but were consistent in three broods of offspring from high maternal Ca. Similar pattern was observed in *D*. *pulex* but neonates born to high maternal Ca significantly reduced from brood Fa to Fc (Fig 3B; *p*<0.05).

### Survival

**Fig 4:**
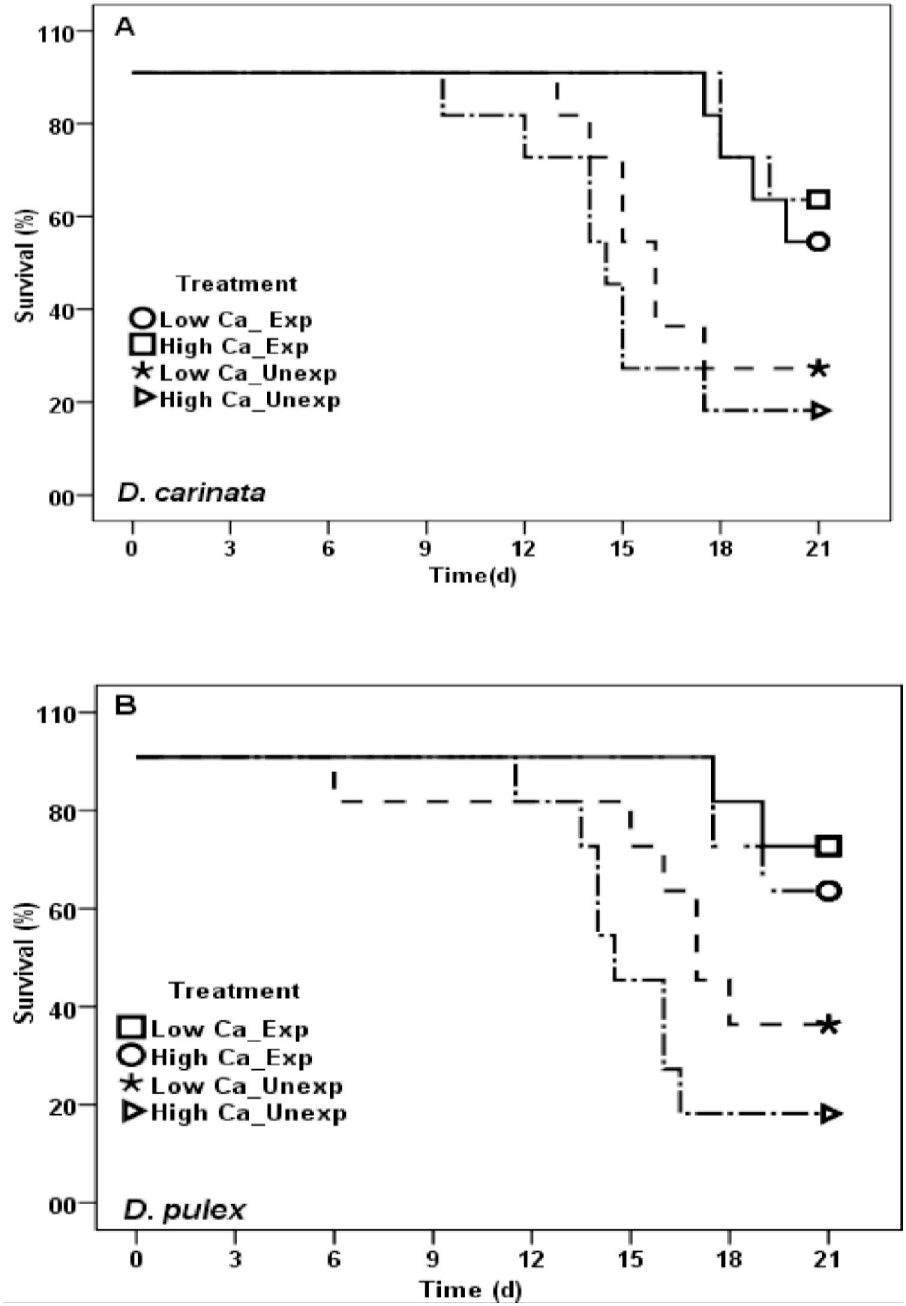
Survival rate of offspring from exposed and unexposed mothers at low and high Ca (mg L^-1^), raised in control COMBO medium. Each data point represents mean of 10 initial replicates.

Survival of offspring was significantly different between exposed and unexposed *D*. *carinata* (Mantel Cox log-rank test, χ2 = 11.25, *p*<0.01), with average survival of 65% and 23% respectively on Day 21 (Fig 4A). Similarly, survival of offspring from exposed *D*. *pulex* was 35% higher than unexposed (Fig 4B; Mantel Cox log-rank test, χ2 = 13.05, *p*<0.005). Survival was not significantly different between offspring from low and high maternal Ca exposed mothers in both *D*. *carinata* (Mantel Cox log-rank test, χ2 = 2.25, *p*>0.05) and *D*. *pulex* (Mantel Cox log-rank test, χ2 = 0.892, *p*>0.05). Similarly, survival in offspring from unexposed *D*. *carinata* (low and high maternal Ca) was not significantly different (Fig 4A; Mantel Cox log-rank test, χ2 = 0.854, *p*>0.05), while significantly different in *D*. *pulex*, and offspring from high maternal Ca has high survival rate than offspring from low maternal Ca (Fig 4B; Mantel Cox log-rank test, χ2 = 5.43, *p*<0.037).

**Fig 5:**
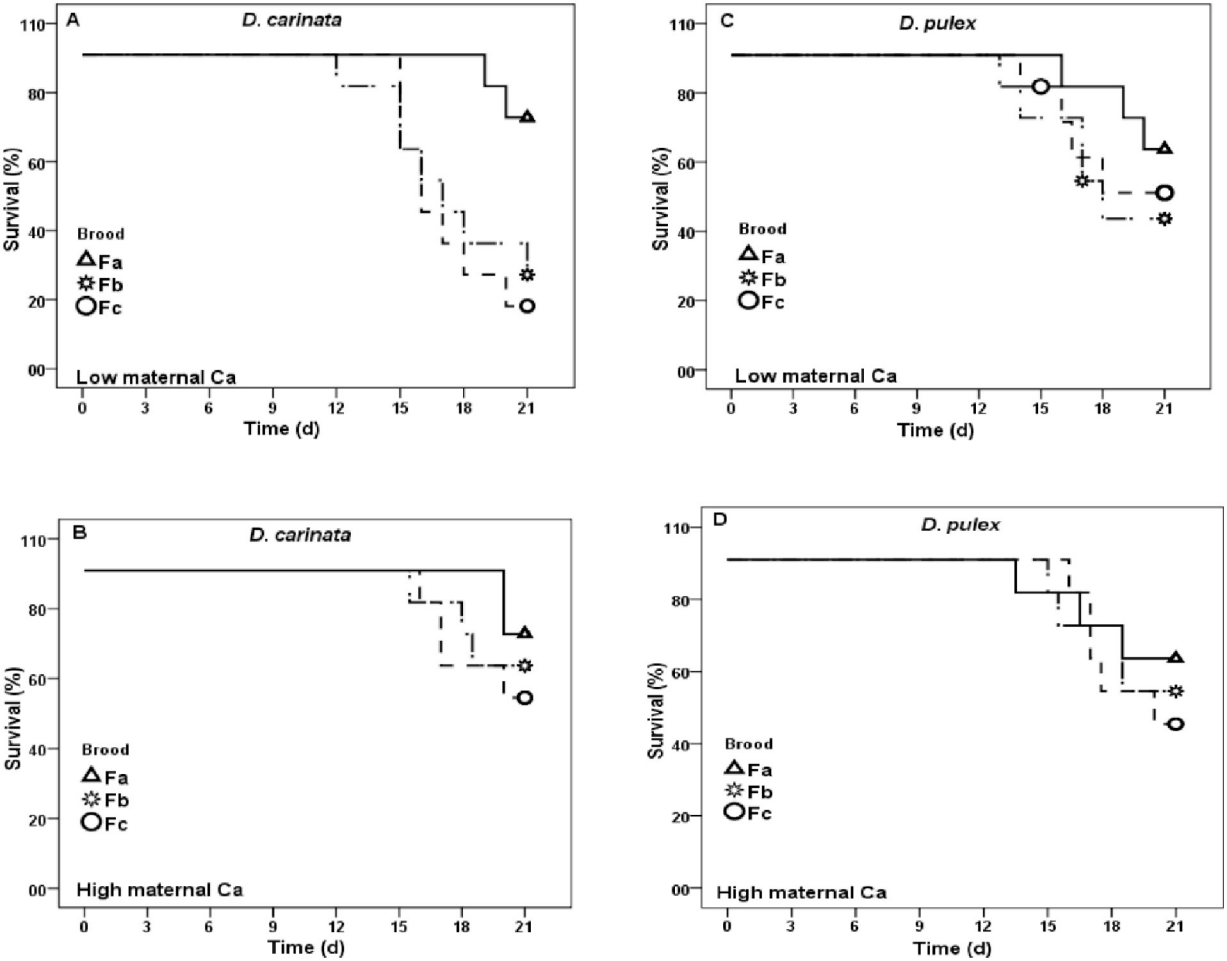
Survival rate of offspring reared in Ca deficient medium (1 mg L^-1^), whose mothers exposed to *M*. *aeruginosa* and either low (2.5 mg L^-1^) (a) or high (10 mg L^-1^) (b) Ca. Each data point represents mean of 10 initial replicates. Fa, Fb and Fc represents third, fifth and seventh brood respectively.

Offspring from exposed mothers, when raised in Ca deficient medium and increasing amount of *M*. *aeruginosa*, survival of low maternal Ca offspring were significantly different among three alternative broods in *D*. *carinta*, while not significantly different in offspring from high maternal Ca mothers (Table 2; Fig 5A and 5B). Survival of *D*. *carinata* in offspring from low maternal Ca was 73% in Fa, and decreased to 27 and 18% in Fb and Fc respectively (Fig 5A). However, percent survival in *D*. *carinata* offspring from high maternal Ca was not significantly different among three broods (Fig 5B). In case of *D*. *pulex*, no significant survival difference was found in offspring from low and high maternal Ca and among broods (Fig 5C and 5D).

**Table 2:** Summary table of Log Rank (Mantel-Cox) test for survival (%) of offspring raised in Ca deficient medium (1 mg L^-1^), whose mothers were exposed to *M*. *aeruginosa* and either low (2.5 mg L^-1^) or high (10 mg L^-1^) Ca.

	Log Rank (Mantel-Cox)			
*Daphnia* species	Maternal Ca	Chi-Square	df	*p*.
***D*. *carinata***	Low	7.42	2	**0.024**
	High	0.934	2	0.627
***D*. *pulex***	Low	1.11	2	0.574
	High	0.496	2	0.781

df, degrees of freedom; *p*, significance of the ANOVA test.

## Discussion

Our results showed the importance of maternal Ca on *Daphnia* inducible defenses in their offspring against *M*. *aeruginosa*. *D*. *carinata* and *D*. *pulex* growth and survival was increased in their offspring through maternal induction. Offspring from exposed *Daphnia* showed markedly enhanced survival and resistance against increasing amount of *M*. *aeruginosa* as compared to unexposed. Offspring of exposed *Daphnia* produced large number of neonates in short time to first brood and small size as compared to unexposed. This supports previous findings that offspring whose mothers experienced toxic *Microcystis* were more resistant than that of inexperienced [13]. Similarly, Gustafsson, Rengefors [10] found that increased fitness of offspring from *D*. *magna* with experienced mothers was usually accompanied by a fast maturation and an increasing number of offspring. Furthermore, we explored the effect of maternal Ca on *Daphnia* induced resistance and its outcome on offspring growth performance. As vital nutrient sources, maternal phosphorous and polyunsaturated fatty acids increase offspring fitness and survival in *D*. *magna* [15, 37]. Riessen, Linley [34] showed that low Ca increase vulnerability of *D*. *pulex* to its predator larvae by reducing *Daphnia* body size and block the kairomone-induced development of adaptive defense. In the present study, offspring of both *Daphnia* species from high maternal Ca and prior *M*. *aeruginosa* experience produce more neonates with large size as compared to that of low maternal Ca. Sakwinska [40] investigated that quality of the maternal environment influences size at birth, number of offspring and energy allocation in offspring. Nutrient stress and maternal environment can strongly affect her offspring fitness and survival [37, 41]. In our first experiment, enough Ca was available in offspring environment, but offspring growth and reproduction decreased whose mothers experienced low maternal Ca. Similarly, Giardini, Yan [42] assessed that high maternal Ca enhance fitness in *D*. *magna* as compared to low Ca. In our second experiment, life history parameters in offspring of high maternal Ca were consistent in three broods in terms of increased growth and reproduction, while maternal effects were not consistent in offspring taken from mothers having low Ca, and life history traits changed. A possible explanation may be that *Daphnia* may expend more energy on Ca uptake, which will be allocated for maternal induction in low Ca environment, or it may involve physiological effect that the *Daphnia* may be unable to transfer proper information to their offspring, which may not persist long under low ambient Ca. This physiological cost or nutrient stress may account for the reduction in induced maternal effects with reduced growth, long maturation time and small body size in offspring whose mothers experienced low Ca. Pajk, von Elert [6] observed that good maternal food quality in *D*. *magna* and *D*. *pulex* was more important under less favourable temperature conditions. Investment in costly defense traits could reduce energy available for investment in other traits [43]. Similarly, Riessen, Linley [34] showed that *D*. *pulex* produced spines at high Ca over 3 instars, while unable to develop large neck spines in second- and third-instar at low Ca concentration. It may be due to the need for extra Ca to produce these defensive adaptations.

Maternal environment shape the physiological regulation of offspring, however, this maternal maintenance may change in fluctuating environment [44]. Offspring experiencing significant environmental changes may ultimately alter the energy for growth and reproduction [45]. We found that life history parameters of offspring, such as time to first brood, number of neonates at first clutch, body length and total population was impacted by maternal Ca, when raised on increased amount of *M*. *aeruginosa*. Here we expect that optimal investment allocation into different fitness components may be dependent on the biotic and abiotic setting and may force the organism to allocate more resources to handle a challenging abiotic environment at the cost of reduced growth and/or reproduction, as we observed in case of offspring from low maternal Ca. Due to increasing incidence of cyanobacterial blooms [4] and decline of Ca concentration in soft-water lakes [29], we expect that their combined effect will negatively influence *Microcystis*-*Daphnia* interactions. Previous research has explored the combined effects of biotic and environmental stressors on the fitness and survival of *Daphnia* and other organisms [27, 31, 46]. *D*. *magna* evolved a faster intrinsic growth rate in monocultures exposed to a 4°C increase in temperature, but evolved smaller size at maturity in microcosm containing competitors, predators and parasites [47]. Under increasing temperature and nutrient loading, the zooplankton will be subject to increasingly intense selection pressure to tolerate cyanobacteria [48, 49]. Similarly, *C*. *vulgaris* under rotifer grazing pressure became smaller in diameter as a defense response, resulting in 32% lower rotifer growth rate relative to the ones feeding on non-defensive algae [50]. Zhu, Wang [51] showed that the defensive response of *S*. *obliquus* induced by exposure to *Daphnia* infochemical was impaired under insufficient Phosphorous and light intensity. Rise in cyanobacterial blooms along with other abiotic factors, such as rise in temperature and CO_2_, as well as UV can negatively affect *Daphnia* growth and survival. However, these functional implications are speculative and require further investigation.

Maternal diet effects becomes weaker with time, and thus effects of maternal provisioning are most pronounced in early developmental stages [52]. Maternal polyunsaturated fatty acids (PUFA) affected offspring growth and survival in *D*. *magna* [15], while the importance of maternal PUFA provisioning may decrease at later stages due to the rapid fatty acid turnover in *Daphnia* [53]. Our results showed that induce tolerance against *M*. *aerugionsa* was decreased in Ca deficient medium in three alternative broods in offspring from low maternal Ca. Time to first brood increased in subsequent 5^th^ and 7^th^ broods as compared to 3^rd^ brood. Similarly, body length and total offspring increase and decrease respectively in three alternative broods, while consistent in offspring from high maternal Ca. Poor maternal diets may have adverse effects on *Daphnia* population and growth, and low concentrations of available Ca may restrict growth, following a molt by limiting the amount of new exoskeleton that can be sufficiently calcified [42]. Additionally, compensatory growth at later life stages can be associated with costs that are only evident much later in adult life [54]. In future, considering multi-broods or multigenerational effects may enhance our understanding of maternal diets on offspring growth and reproduction.

In *Daphnia*, intraspecific variation in tolerance represents a high potential for micro-evolutionary responses to increased cyanobacterial abundance [36]. However, this micro-evolution may be affected by other abiotic factors such as warming and eutrophication [55]. We found variations in life history traits and survival in *D*. *carinata* and *D*. *pulex*. For instance, offspring from exposed mothers when grown in Ca deficient medium, neonates produced at first clutch in three alternative broods successively decreased in *D*. *pulex* (offspring from high maternal Ca), while remain consistent in *D*. *carinata*. Similarly, survival was reduced in fifth and seventh broods as compared to third in low maternal Ca offspring in *D*. *carinata*. In contrast, *D*. *pulex* survival was almost the same in offspring from low and high maternal Ca and among three alternative broods. This contrast may be due to species specific Ca requirement. Previously, various studies investigated that different *Daphnia* species and population has different Ca requirements [28, 56]. Adult *Daphnia* Ca requirement is less than juvenile due to different functional requirements, as juveniles invest in growth and calcification, while adults has the extra burden of reproduction [27, 42]. Similar other studies also showed the presence of extensive genetic variation in the life-history responses of *Daphnia* [46, 57]. We expect that Ca decline may decrease *Daphnia* resistance and abundance against toxic *Microcystis* and might enhance blooms, but it can’t be generalize due to species specific Ca requirements and need further studies. Prepas, Pinel-Alloul [58] investigated that Ca decline weaken phosphate-binding capacity of lake sediments which enhance cyanobacterial growth. Considering, Riessen, Linley [34] study, that low Ca disable *D*. *pulex* defense against predator, we expect that reduce population and survival in low Ca environment may further keep *Daphnia* more vulnerable in rising cyanobacterial blooms.

A commonly used proxy for fitness is the potential of organism to survive and deliver average number of offspring to next generation [59]. In addition to offspring density, body size has been recognized as an important factor in aquatic ecosystem, and competitive interactions for resources promote diversity in aquatic communities by size [60]. Low Ca concentrations directly reduce *Daphnia* body size with an apparent Ca threshold at or slightly above 1.5 mg/L [34]. We found a contrast in maternal and offspring Ca availability on offspring life history traits and fitness parameters. In experiment 1, offspring from exposed mothers having low Ca, raised on diet of increased amount of *Microcystis* and sufficient Ca, produced smaller size individuals and reduced population. Similarly, offspring from low maternal Ca when reared in Ca deficient medium, produced large neonates with reduced population in fifth and seventh broods as compared to third brood, while high maternal Ca offspring produced small size and increased number of total offspring. In addition, time to first brood prolonged nearly one day in subsequent broods in offspring from low maternal Ca in both *Daphnia* species. The net energy available for growth and reproduction will be reduced by large size and may act as a stronger constraint as Ca decline. Moreover, the reproduction may therefore become more costly for larger *Daphnia* at reduced Ca and may be more vulnerable to increasing amount of *Microcystis* and therefore, reduced *Daphnia* population. Low food concentration can result in reduce population with large size of neonates [61]. Similarly, Garbutt, Scholefield [62] observed that maternal food affect offspring feeding rate and body size. A potential additional explanation for larger size could be an effect of prolong time to first brood in offspring from low maternal Ca as compared to high maternal Ca.

We have shown that maternal effects enhance growth and survival of *Daphnia* against *Microcystis*. Our finding further adds that high maternal Ca increased offspring growth and survival in increasing amount of *M*. *aeruginosa*. We expect that increasing amount of *Microcystis* and declining Ca could increase the loss of large daphniids, a key herbivore in the aquatic food web. Large-scale lake surveys and controlled field experiments will be useful to predict the interacting effects of cyanobacteria and declining Ca on *Daphnia* growth and survival. Therefore, we encourage future research with more species and clones of *Daphnia* to confirm the generality of our findings.

## Supporting information

S1 Table *p* values for Fig 3, values in bold are significantly different (*p<0*.*05*). (DOCX)
