# Supplementary material for: The importance of calcium in improving resistance of Daphnia to Microcystis
Source: PLoS One. 2017 Apr 17;12(4):e0175881. doi: 10.1371/journal.pone.0175881 (PMC5393884; doi:10.1371/journal.pone.0175881)
Supplement: S1 Table — (DOCX) [file pone.0175881.s001.docx]

| Parameters | *Daphnia* species | Maternal Calcium | Broods | *p* |
| --- | --- | --- | --- | --- |
| Time to first brood (d) | *D. carinata* | Low | Fa x Fb | **<0.000** |
|  |  |  | Fa x Fc | **<0.000** |
|  |  |  | Fb x Fc | 0.701 |
|  |  | High | Fa x Fb | 0.646 |
|  |  |  | Fa x Fc | 0.567 |
|  |  |  | Fb x Fc | 0.732 |
|  | *D. pulex* | Low | Fa x Fb | 0.933 |
|  |  |  | Fa x Fc | **<0.000** |
|  |  |  | Fb x Fc | **<0.000** |
|  |  | High | Fa x Fb | 0.856 |
|  |  |  | Fa x Fc | 1.00 |
|  |  |  | Fb x Fc | 0.934 |
| Neonates at first clutch (n) | *D. carinata* | Low | Fa x Fb | 1.00 |
|  |  |  | Fa x Fc | 0.938 |
|  |  |  | Fb x Fc | 0.540 |
|  |  | High | Fa x Fb | 0.657 |
|  |  |  | Fa x Fc | 0.497 |
|  |  |  | Fb x Fc | 0.632 |
|  | *D. pulex* | Low | Fa x Fb | 0.765 |
|  |  |  | Fa x Fc | 0.672 |
|  |  |  | Fb x Fc | 0.543 |
|  |  | High | Fa x Fb | **0.024** |
|  |  |  | Fa x Fc | **<0.000** |
|  |  |  | Fb x Fc | **0.001** |
| Number of molts (n) | *D. carinata* | Low | Fa x Fb | 0.426 |
|  |  |  | Fa x Fc | 0.975 |
|  |  |  | Fb x Fc | 1.00 |
|  |  | High | Fa x Fb | 0.754 |
|  |  |  | Fa x Fc | 0.845 |
|  |  |  | Fb x Fc | 1.00 |
|  | *D. pulex* | Low | Fa x Fb | **0.007** |
|  |  |  | Fa x Fc | **0.001** |
|  |  |  | Fb x Fc | 0.825 |
|  |  | High | Fa x Fb | 0.723 |
|  |  |  | Fa x Fc | 0.871 |
|  |  |  | Fb x Fc | 0.597 |
| Average size (mm) | *D. carinata* | Low | Fa x Fb | **0.009** |
|  |  |  | Fa x Fc | **<0.000** |
|  |  |  | Fb x Fc | **0.036** |
|  |  | High | Fa x Fb | 0.826 |
|  |  |  | Fa x Fc | 0.967 |
|  |  |  | Fb x Fc | 1.00 |
|  | *D. pulex* | Low | Fa x Fb | 0.600 |
|  |  |  | Fa x Fc | **<0.000** |
|  |  |  | Fb x Fc | **<0.000** |
|  |  | High | Fa x Fb | 0.496 |
|  |  |  | Fa x Fc | 0.363 |
|  |  |  | Fb x Fc | 0.238 |
| Total offspring (n) | *D. carinata* | Low | Fa x Fb | **<0.000** |
|  |  |  | Fa x Fc | **<0.000** |
|  |  |  | Fb x Fc | **0.006** |
|  |  | High | Fa x Fb | 0.840 |
|  |  |  | Fa x Fc | 0.786 |
|  |  |  | Fb x Fc | 0.934 |
|  | *D. pulex* | Low | Fa x Fb | **<0.000** |
|  |  |  | Fa x Fc | **<0.000** |
|  |  |  | Fb x Fc | **<0.000** |
|  |  | High | Fa x Fb | 0.913 |
|  |  |  | Fa x Fc | 0.767 |
|  |  |  | Fb x Fc | 0.712 |

**Table S1. *p* values for Fig 3, values in bold are significantly different (*p<0.05).***
